# Do Interventions to Increase Walking Work? A Systematic Review of Interventions in Children and Adolescents

**DOI:** 10.1007/s40279-015-0432-6

**Published:** 2015-12-01

**Authors:** Angela Carlin, Marie H. Murphy, Alison M. Gallagher

**Affiliations:** Northern Ireland Centre for Food and Health, Biomedical Sciences Research Institute, University of Ulster, Coleraine Campus, Cromore Road, Coleraine, BT52 1SA UK; Centre for Physical Activity and Health, Sports and Exercise Sciences Research Institute, University of Ulster, Newtownabbey, UK

## Abstract

**Background:**

Physical activity (PA) levels decline as children move into adolescence, with this decline more notable in girls. As a consequence, many young people are failing to meet current PA guidelines. Walking has been a cornerstone of PA promotion in adults and may provide an effective means of increasing PA levels among younger people.

**Objective:**

Our objective was to conduct a systematic review of interventions aimed at promoting increased levels of walking among children and adolescents.

**Methods:**

Eight electronic databases—CINAHL, Cochrane Library CENTRAL database, EMBASE, Medline OVID, PsycINFO, Scopus, SPORTDiscus and Web of Knowledge—were searched from their inception up to January 2015 using predefined text terms: walking terms AND intervention terms AND population terms AND (physical activity OR exercise). Reference lists of published systematic reviews and original articles included in the review were also screened. Included studies were randomised and non-randomised controlled trials reporting a specific measure of walking levels (self-reported or objective) to assess the effectiveness of interventions aimed at promoting walking in children and adolescents (aged 5–18 years). Only full articles published in English in peer-reviewed journals were included. Risk of bias and behaviour change techniques of included studies were assessed.

**Results:**

Twelve studies were included in this review. The majority of studies assessed interventions delivered within an educational setting, with one study conducted within the family setting. Nine of the included studies reported significant increases in walking in intervention groups versus controls. Commonly employed behaviour change techniques within successful interventions included goals and planning, feedback and monitoring, social support and repetition and substitution.

**Conclusions:**

Walking interventions, particularly those conducted in the school environment, have the potential to increase PA in children and adolescents. Conclusions on which interventions most effectively increased walking behaviours in this population were hindered by the limited number of identified interventions and the short duration of interventions evaluated. The short-term effectiveness of the majority of included studies on levels of walking in this population is promising and further research, particularly within non-educational settings and targeted at sub-groups (e.g. adolescent girls and overweight/obese children and adolescents), is warranted.

**Electronic supplementary material:**

The online version of this article (doi:10.1007/s40279-015-0432-6) contains supplementary material, which is available to authorized users.

## Key Points

**Table:** 

Walking may present a feasible means of increasing physical activity participation among children and adolescents.
Walking interventions, delivered in the school setting, may be effective in increasing physical activity.
The evidence base is limited by the small number of studies conducted to date in this population and by the short-term duration of included studies.

## Introduction

Low levels of physical activity (PA), coupled with increased time spent being inactive or engaged in sedentary behaviours, are thought to be key contributors in the onset of obesity [1]. Consequently, the promotion of PA amongst children and adolescents is justifiably at the forefront of campaigns and policies to tackle childhood obesity [2, 3].

In addition to the role of PA in energy balance [1], regular PA has been associated with a number of health benefits for children and adolescents, including improvements in skeletal health, mental well-being and indicators of cardiorespiratory and metabolic health [4, 5]. Higher levels of PA [6] and, more specifically, moderate to vigorous intensity physical activity (MVPA) [7, 8] has been associated with improvements in cardiometabolic risk factors, as well as several positive mental health outcomes such as lower levels of anxiety and depression in adolescents [9].

Within the UK, it is recommended that children and young people (aged 5–18 years) should engage in at least 60 min MVPA each day [10]. However, a considerable proportion of children and young people are failing to meet current PA recommendations [11, 12]. Approximately 51 % of children aged 7 years were reported to be meeting PA guidelines in the UK [12]. PA levels tend to decline as children move into adolescence, with girls participating in less PA than boys [13]. While the rate of decline is similar amongst sexes, the age at which these decreases are observed occurs earlier in girls [13, 14]. Recent data from a 10-year cohort study highlighted the decline in PA as children move into adolescence, with total PA falling by approximately 30 % in girls and 20 % in boys from ages 9 to 15 years [15].

Several authors have highlighted the potential for youth PA to track into adulthood and predict future PA levels [16, 17], with evidence showing a stronger tracking of PA in boys [16, 18]. The promotion of PA amongst children and adolescents is therefore pertinent since PA during childhood and adolescence may provide a base for future PA habits [19], with those partaking in regular PA during adolescence more likely to maintain these activity patterns into adulthood [17]. Coupled with the potential tracking of childhood overweight into adulthood [20], this stage of the lifecycle represents a key period for the promotion of PA behaviours. The development of effective interventions to promote PA in children and adolescents is a key research priority. However, given the differences in observed PA levels of children and adolescents, and the different factors that can influence PA at these two stages of the lifecycle [21], there is a need to consider the potential effectiveness of interventions on PA in these two populations separately.

Walking has been described as the most natural form of PA [22], and can provide a practical, inexpensive option for children and adolescents to increase physical activity [23]. Its relative ease of adoption and lower skill requirement compared with other activities or sports make it particularly suited to overweight and obese children and young people [23]. Walking provides an ideal foundation for the most sedentary to move towards increased PA [22] with the advantage of being a convenient form of activity; it requires no specialist skills to participate and has little or no economic cost to the individual [22], requiring little equipment and resources as compared with other forms of PA. Promoting increased levels of brisk walking has been cited as a most feasible means of helping people meet current PA guidelines [24, 25], with levels of adoption likely to be consistent irrespective of age, sex and ethnicity, as well as socioeconomic status [24]. Walking at a speed of approximately 5 km/h is considered moderate intensity PA for most adults [26], although the application of these cut-offs in younger populations is less clear, with suggestions that walking speeds of 3–4.5 km/h equates to moderate intensity PA in children [27, 28]. Additionally, as in adults, brisk walking can be easily accumulated in short bouts spread throughout the day making it a flexible, unstructured form of PA which may appeal to this population [29] and contribute to the MVPA guidelines in children [30]. In addition to the benefits of walking in helping populations meet current PA guidelines, promoting walking has far-reaching benefits in relation to climate change and sustainable transport [25]. Consequently, walking forms a key component of regional schemes to promote active travel to school [31], with evidence highlighting that boys who walk to school also have higher levels of PA after school [32].

Walking may provide low active adolescents, in particular females, with a form of activity that overcomes frequently cited barriers to PA participation; for example, wearing uncomfortable uniforms, feeling marginalised during school time PA and not enjoying competitive sports with an emphasis on winning [33]. Instead, walking offers a means of overcoming these barriers and can encompass some of the facilitators of PA in this age group; for example, active time can be shared with friends, with activity that is fun and informal in nature [29, 33]. As such, interventions which promote walking in subgroups of the population with lower levels of PA, for example adolescents, may be an effective means of offsetting the age-related decline in PA.

Whilst a number of systematic reviews have examined the effects of interventions targeted at increasing levels of PA in children and adolescents [34, 35], none have looked specifically at walking interventions in this population. A previous review of walking interventions, involving mainly adult populations, concluded that interventions could increase walking by up to 30–60 min per week when targeted at participants who were sedentary or motivated to change [36]. However, changes in walking levels were less notable in the long-term [36]. Within these systematic reviews, the identification of behaviour change techniques (BCTs) that may have contributed to positive changes in PA-related outcomes was not included as an objective, making it difficult to judge which BCTs are likely to have been successful within the intervention content, which could then be utilised in future intervention design [37, 38]. The development of the BCT taxonomy [39] has provided a tool to investigate the use of BCT in health behaviour change. To date, the taxonomy has been used to identify effective intervention components to promote PA [38], and more specifically, cycling and walking [40]. A number of BCTs, including self-monitoring and intention formation, have been identified as successful and warrant inclusion in future interventions to promote cycling and walking [40]. This review focused on adult-only studies and thus there is limited evidence on what BCTs are most effective in children and adolescents.

Given the declining levels of PA in children and adolescents, and the acceptability of walking as a form of activity, a review of the effectiveness of walking interventions in this population is warranted. The aim of this systematic review was to evaluate the effects of interventions aimed at promoting levels of walking among children and adolescents on subsequent walking behaviours. Within this, specific objectives included comparing the effects of interventions aimed at children and adolescents and the settings in which these interventions are delivered, identifying the frequency of BCT use within interventions with desirable/undesirable outcomes and comparing the sex differences for walking interventions in children and adolescents.

## Methods

The methodology for this review was based on the Cochrane Systematic Review Methodology [41] and Preferred Reporting Items for Systematic Reviews and Meta-Analysis (PRIMSA) [42]. A protocol for this systematic review has not been previously published.

### Search Strategy

Electronic databases were searched up to 7 January 2015 using text terms with appropriate truncation and medical subject headings (MeSH). The following databases were searched: CINAHL (1934–2015), Cochrane Library CENTRAL database, EMBASE (1980–2015), Medline OVID (1946–2015), PsycINFO (1806–2015), Scopus (1966–2015), SPORTDiscus (1984–2015) and Web of Knowledge (1900–2015). Timespan limits were not imposed to ensure that no reviews in this area had been previously published. The search filter was walking terms AND intervention terms AND population terms AND (physical activity OR exercise). To ensure the sensitivity of the search in identifying all potentially relevant papers, the search filter was developed and tested in Medline OVID (see Electronic Supplementary Material, Table S1 for search criteria) and adapted as appropriate for each database. The reference lists of published relevant systematic reviews [34–36, 43–45] and original articles included in this review were also screened to identify additional potentially relevant articles.

### Eligibility Criteria

Studies were included in this review if all of the following criteria were fulfilled: (i) an intervention in which the main component, or one of the main components, was aimed at increasing walking behaviour, (ii) a control or minimal intervention group was included, (iii) the mean age of participants was between 5 and 18 years, (iv) one of the main study outcomes was a measure of walking levels (self-reported or objective) and (v) the effect of this outcome was available at baseline and at least one other time point.

A limit was imposed on all databases to search for human studies only. Only full articles published in English in peer-reviewed journals were included. To ensure all relevant intervention studies were included to fully address the purpose of this systematic review, this review was not limited to randomised controlled trials (RCTs). Although RCTs provide the strongest evidence on the effectiveness of interventions, such study design may not be feasible within certain settings [35], and the exclusion of non-randomised trials may lead to potentially successful interventions being overlooked, which can be replicated with a stronger study design to improve their methodological quality [35]. Studies were excluded if the control group was exposed to any intervention also likely to bring about changes in walking behaviour. In addition, walking interventions that were targeted at a study population with a diagnosed medical condition, such as diabetes mellitus, were excluded.

### Study Selection and Data Extraction

Titles and abstracts of potentially relevant articles were screened by a single reviewer (AC). Duplicates were removed and articles that did not meet the inclusion criteria were excluded. Full texts were assessed for eligibility by two reviewers (AC, AG) and any articles which were ambiguous regarding inclusion were independently assessed by all members of the research team against the eligibility criteria (AC, AG, MM). Disagreements regarding inclusion of ambiguous articles were discussed with all members of the research team and a consensus was agreed. A pre-designed data extraction form was used to collate data from individual studies, including country/setting, study design, method of randomisation, inclusion criteria, baseline characteristics of participants, representativeness of the study sample, details on the intervention and control conditions to include information on attrition rates within each study, length of follow-up, PA-related outcome measures and the methods used to collect PA data, secondary outcome measures, results and where available, information on cost effectiveness and adverse outcomes as a result of the intervention. Data for each included study were extracted by a single reviewer (AC) and were checked by a second reviewer (AG).

### Change in Walking Behaviour

Data on outcomes measures related to changes in walking were extracted for all studies. The effectiveness of an intervention was classed as desirable if the intervention group significantly increased their walking compared with controls.

### Risk of Bias

Risk of bias was assessed for each included study using Cochrane methodology [41]. Risk of bias was assessed independently by two members of the research team (AC, MM). Results were compared and where consensus could not be reached, a third member of the research team was consulted (AG).

### Behaviour Change Techniques

All methods to change walking behaviour within the interventions were coded using the BCT taxonomy, a methodological tool for reliable and systematic identification of intervention content [39]. This allows the identification of components within health interventions that are effective and which can then be replicated and implemented across disciplines. Each intervention was independently coded by two authors (AC, MM) and disagreements were resolved through discussion.

## Results

### Study Selection

**Fig. 1:**
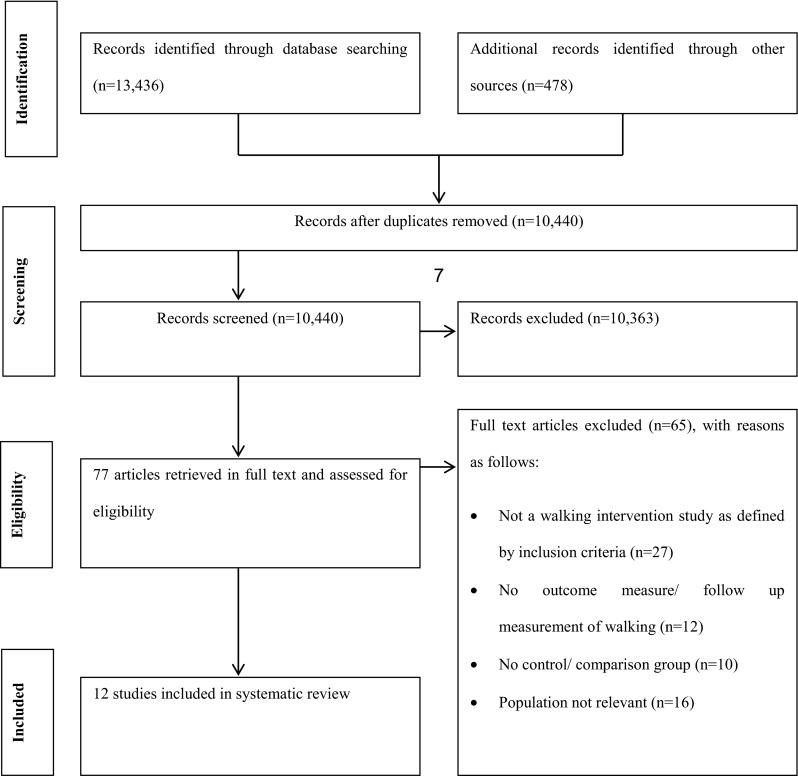
PRISMA flow diagram of literature search for walking interventions in children and adolescents [42]

The search identified 10,440 references which were screened by abstract for eligibility (see Fig. 1). Of these, 10,363 references were eliminated as they did not meet the selection criteria, namely (i) not the relevant population, (ii) not an intervention or (iii) not related to walking. Subsequently, 77 full-text articles were retrieved and assessed for eligibility. In total, 12 studies were included in this review.

### Characteristics of Included Studies

The characteristics of included studies are detailed in Table 1. The 12 studies within this review involved a total of 3702 children and adolescents. Nine studies included children (aged 5–12 years), with three studies targeted at adolescents (aged 13–18 years). Ten studies were mixed sex, with two studies targeted at a single sex (females). The majority (*n* = 9) of included studies used objective measures to assess changes in walking (pedometers, accelerometers or mapping technology) while others (*n* = 3) used direct observation or survey methods. Follow-up times for the measurement of walking-related outcomes ranged from 7 days to 18 months within interventions. Only one included study evaluated the promotion of walking outside the educational setting (school or college). The majority of included studies (75 %) were conducted in the US (*n* = 5) or the UK (*n* = 4).

**Table 1:** Intervention characteristics of included studies

Study	Design; country	Setting (delivered by)		Participants	Outcome measures	Follow-up^a^	Intervention description	Main results
Studies in children (aged 5–12 years)								
Sirard et al. [46]	RCT (individual); USA		Active travel to school (researcher)	*n* = 11 Mean age: I group 9.7 y (SD 0.90) C group 9.5 y (SD 1.02) 55 % male	Accelerometer (counts/min, % of time spent in MVPA)	2 weeks	I: Walking school bus, walked at normal pace C: Morning commute was not altered	Counts/min and % time MVPA: Before school* GCT** All days ↔ Weekdays ↔
McKee et al. [55]	Quasi-experimental controlled trial (school); UK		School and family (teacher)	*n* = 55 Mean age 9 y 40 % male	Distance travelled to school by walking (m)	10 weeks	I: School-based active travel project delivered through teacher handbooks and pupil packs C: No intervention	Distance walked***
McMinn et al. [47]	Quasi-experimental controlled trial (school); UK		Active travel to school (teacher)	*n* = 166 Mean age: I group 8.7 y (SD 0.51) C group 8.6 y (SD 0.48) 60 % male	Accelerometer (daily step count)	6 weeks	I: School-based active travel project delivered through teacher handbooks and pupil packs C: No intervention	Daily step count ↔
Bungum et al. [48]	Quasi-experimental controlled trial (school); USA		Active travel to school (teacher/school)	*n* = 172 Aged 5–11 y 53 % male	Observation: mode of travel to school (number of walkers)	1 week, 2 weeks	I: School participated in NMD (encourages active travel to school) C: School did not participate in NMD	Number of walkers: 1 week ↔ 2 weeks ↔
Mendoza et al. [49]	Quasi-experimental controlled trial (school); USA		Active travel to school (researcher)	*n* = 653 Aged 5–11 y 49 % female Low income school, urban community	Mode of travel to school: show of hands	1 month, 6 months, 12 months	I: Walking school bus to and from school up to 5 days/week C: Received standard information on school transportation (no intervention)	Walking to school: 1 month** 6 months** 12 months***
Mendoza et al. [50]	Cluster RCT (school); USA		Active travel to school (researcher)	*n* = 149 Mean age 9.7 y (SD 0.7) 70 % male Low income schools	Accelerometer (mins/day of MVPA)	4/5 weeks	I: Walking school bus to and from school up to 5 days/week C: Received standard information on school transportation (no intervention)	Minutes/day MVPA*
Wen et al. [51]	Cluster RCT (school); Australia		Active travel to school (teacher/school/local council)	*n* = 1996 Aged 10–12 y 53 % female	Student: 5-day travel mode survey Parent: mode of travel for typical week (walking trips)	18 months	I: Multicomponent active travel to school, provided resources for students, schools and parents C: Received a programme on healthy eating at school	Walking to school: Student survey ↔ Parent survey*
Ford et al. [53]	RCT (individual); UK		School only (teacher/researcher)	*n* = 152 Aged 5–11 years 52 % male	Accelerometer (weekday counts 0900–1500 hours)	15 weeks	I: Participated in an accumulated brisk walking programme: 2 × 15 min walking sessions, at least three times/week C: Normal, seated lessons during walking (no intervention)	Mean weekday counts^b^: I group***
Morrison et al. [57]	RCT (individual); UK		Home (family/researcher)	*n* = 30 Mean age 10.9 y 67 % female	Accelerometer (total PA, % of time in sed, LPA, MVPA, time sitting)	11 weeks	I: Family-based dog walking intervention, BCTs employed to increase frequency, intensity and duration of dog walking C: Did not receive any information, asked to continue normal routine	Total PA ↔ % Sed ↔ % LPA ↔ % MVPA ↔ % time sitting ↔
Studies in adolescents (aged 13–18 years)								
Schofield et al. [54]	Quasi-experimental controlled trial (school); Australia	School only (researcher)		*n* = 68 Mean age 15.8 y (SD 0.8) Girls only	Pedometer (4-day step count)	6 weeks, 12 weeks	I: 6 weekly sessions and 6-week maintenance phase I group 1: pedometer-based self-monitoring, encouraging step increases up to 10,000/day I group 2: self-monitoring through recording minutes of daily activity, daily minute prescriptions encouraging increases up to 30–60 mins/day C: Did not attend weekly meetings	4-day step count: 6 weeks Group 1 ↔ Group 2 ↔ 12 weeks Group 1* Group 2 ↔
Shimon and Petlichkoff [56]	RCT (class); USA	School only (teacher)		*n* = 113 Mean age 13.2 y (SD 0.72) 54 % male	Pedometer (daily step count)	5 weeks	I group 1: Self-regulation strategies: unsealed pedometers for self-monitoring; plotted daily steps; information on goal setting strategies I group 2: Unsealed pedometers, recorded daily steps C: Wore sealed pedometers	Daily step count: Group 1* Group 2↔
Lee et al. [52]	RCT (class); Taiwan	School only (nursing tutor/researcher)		*n* = 91 Mean age 16.2 y (SD 0.41) Girls only	Pedometer (steps/day of aerobic walking)	12 weeks	I: Instructed to complete 12,000 steps and/or 60 mins aerobic walking/day; individual discussions, goal setting, given pedometers C: Usual content and activity of physical education classes	Aerobic walking**

*C* control, *BCTs* behaviour change techniques, *GCT* general commute time, *I* intervention, *LPA* light physical activity, *MVPA* moderate-to-vigorous physical activity, *NMD* Nevada Moves Day, *PA* physical activity, *RCT* randomised controlled trial, *SD* standard deviation, *Sed* sedentary

*** *p* ≤ 0.001, ** *p* ≤ 0.01, * *p* ≤ 0.05, ↔ no significant effect between I and C

^a^Data available in relation to changes in walking behaviour

^b^Between-groups effect not reported

In studies where active commuting to school via walking was the target PA behaviour, control groups were instructed to continue with their usual commuting habits [46–48], provided with standard information on school transportation [49, 50] or participated in a healthy eating programme at school while the active travel intervention was delivered [51]. Other interventions detailed that participants continued with their usual structured physical education (PE) classes [52] or participated in seated classroom work while the intervention was delivered [53]. Since no studies incorporated economic evaluations of the interventions, it was not possible to draw conclusions on the cost effectiveness of included studies.

### Effect of Intervention on Walking Levels

All included studies reported walking-related outcome measures at baseline and at least one other time point. The majority of included studies presented this data for the whole sample with only one study reporting this outcome for a subsample (80 %) of participants [53]. Changes in walking behaviour for studies included in the review are presented in Table 1. Interventions were described as desirable if they resulted in significant increases in walking levels between groups at follow-up. Eight of twelve studies [46, 49, 50, 52–56] reported desirable outcomes at follow-up. An active travel to school intervention reported mixed outcomes at follow-up; changes in walking based on self-reported walking by children found no changes at follow-up, while parents reported a desirable intervention effect at follow-up [51]. Undesirable intervention outcomes (i.e. no significant increases in walking) were reported in two active commuting studies [47, 48] and in a family-based dog-walking intervention [57].

A few studies that reported desirable intervention effects on walking levels also reported findings on other health-related outcomes [52–54], with one study reporting positive changes in anthropometric outcomes at follow-up [53]. Based on the small number of studies available, it was not possible to draw conclusions on the associations between walking and other health indices beyond changes in walking behaviour.

### Effect of Interventions in Children (Aged 5–12 years)

Of the nine studies in children, eight evaluated walking interventions in the school setting. Two educational interventions encouraging active travel to school through classroom lessons and interactive resources for families, and targeted at both sexes, reported contrasting outcomes at follow-up [47, 55]. Those participating in the *Travelling Green* project significantly increased their mean distance travelled to school by walking by 574 m [55]. A further study evaluating the *Travelling Green* project, involving different subjects, observed decreases in mean daily steps for both intervention and control [47].

Three walking school bus interventions (all mixed-sex) found increased walking following intervention [46, 49, 50]. Significant differences between intervention and control participants were noted for percentage of time spent in MVPA, both in total time before school (5:00 am–8:09 am) and general commute time (7:25 am–8:09 am), with intervention participants achieving an increase of 25 and 30 % more time spent in MVPA, respectively [46]. A 12-month follow-up of a walking school bus resulted in significant increases in the proportion of children travelling to school by walking (increase of 25 %), assessed by a daily ‘hands-up’ survey [49]. A similar study objectively evaluated PA-related outcomes using accelerometers, with intervention participants significantly increasing their minutes/day of MVPA by 2.2 min at time point 2, compared with a decrease of 4.8 min observed in control participants [50].

A 2-year multi-component active travel to school intervention, targeted at both sexes, assessed changes in walking behaviour using both a student and a parent survey, with significant changes between intervention and control participants evident only within the parent survey [51]. Based on parent data, the percentage of students walking to school significantly increased by 28.8 % in intervention schools [51]. An active travel to school day observed no effect in the number of boys or girls walking to school [48].

In contrast, another mixed-sex school-based intervention which provided regular, structured walks throughout the school day reported significant differences in school time PA in intervention versus control groups [53]. Intervention participants increased their mean daily PA levels during school time by 136.6 counts per minute (cpm) compared with 37.8 cpm in control participants [53]. Only one study evaluated an intervention delivered outside of the educational setting in children (boys and girls), involving a family-based dog walking intervention, with no significant differences reported [57].

### Effect of Interventions in Adolescents (Aged 13–18 years)

All of the studies targeted at adolescents used pedometers as part of the overall intervention content, to promote self-monitoring and provide feedback on behaviours [52, 54, 56]. Pedometers were also used to measure the main outcomes in these studies but were sealed for baseline and follow-up measurements [52, 54, 56], with all three studies in adolescents reporting desirable intervention outcomes. An intervention targeted at junior high school students (mixed sex) was delivered as part of existing PE classes and involved promoting self-regulation amongst participants [56]. Those in the self-regulation group significantly increased their daily steps by 2071–4141 steps/day more than the control group [56]. The study did not observe any significant differences between sexes for daily steps throughout the intervention [56].

The *Girls Stepping Out Program* targeted low-active adolescent girls and involved participants attending 12 weekly group sessions, with participants working towards daily step goals or time-based goals [54]. Those in the pedometer group significantly increased their 4-day step count at follow-up (40,992 steps) compared with controls (34,221 steps) [54]. Finally, a study also targeted at adolescent girls provided participants with daily step targets, with the mean difference in aerobic steps/day significantly higher for intervention (+371 steps/day) compared with control group (−108 steps/day) [52].

### Risk of Bias

#### Low Risk of Bias

Attrition bias was low in eight of the included studies (Table 2). Similarly, reporting bias was low in eight studies, with these studies reporting all expected outcomes. Selection bias was low in five included studies, indicating that random sequence generation had been performed and was adequately described within the studies.

**Table 2:** Risk of bias assessment within studies

Study	Random sequence generation	Allocation concealment	Blinding of participants and personnel	Blinding of outcome assessment	Incomplete outcome data	Selective reporting	Other bias
Sirard et al. [46]	Low	Unclear	High	Unclear	Low	Unclear	High
McKee et al. [55]	High	High	Unclear	Unclear	Low	Low	High
McMinn et al. [47]	High	Unclear	Unclear	Unclear	Unclear	Low	High
Bungum et al. [48]	High	Unclear	Unclear	Unclear	Unclear	Unclear	High
Mendoza et al. [49]	High	High	Unclear	Unclear	Low	Low	High
Mendoza et al. [50]	Low	Unclear	High	Low	Low	Low	High
Wen et al. [51]	Low	High	High	Low	Unclear	Low	High
Ford et al. [53]	Low	Unclear	Unclear	Unclear	Unclear	Unclear	High
Morrison et al. [57]	Low	Low	Unclear	Low	Low	Low	Low
Schofield et al. [54]	High	Unclear	Unclear	Unclear	Low	Low	High
Shimon and Petlichkoff [56]	Unclear	Unclear	Unclear	Unclear	Low	Low	High
Lee et al. [52]	Unclear	Unclear	High	Low	Low	Unclear	High

#### High Risk of Bias

Selection bias was high in five included studies, attributed to these studies allocating participants to study conditions (i.e. intervention or control) in a non-randomised manner. The majority of included studies (*n* = 11) scored highly for other risks of bias. Reasons for this included possible intervention contamination as participants in intervention and control groups were recruited from the same school/college, baseline differences between groups at baseline and incorrect analysis of cluster randomised controlled trials.

#### Unclear Risk of Bias

Performance bias and detection bias were classed as unclear for eight included studies, which was mainly attributed to poor reporting of blinding of outcome assessors to study measures. In addition, many papers failed to adequately report whether participants or study personnel were blinded to group allocation. Selection bias was also classed as unclear for eight included studies, with these studies failing to provide detail on how concealment of allocation to study groups (i.e. intervention or control) was performed.

### Behaviour Change Techniques

Table 3 details the standardised categories of intervention components in accordance with the BCT taxonomy [39]. Eleven categories of BCTs were employed across interventions. The most commonly employed BCTs within interventions reporting desirable outcomes in relation to walking were social support (*n* = 6 studies), repetition and substitution (*n* = 6 studies), feedback and monitoring (*n* = 5 studies) and goals and planning (*n* = 4 studies). Additional BCTs used included shaping knowledge, comparison outcomes, natural consequences, antecedents, associations and rewards and threat. Only one study reporting a desirable intervention outcome did not combine feedback and monitoring with goals and planning [51]. Goals and planning (*n* = 2 studies) and feedback and monitoring (*n* = 2 studies) were also employed within studies reporting undesirable outcomes at follow-up [47, 57].

**Table 3:** Intervention components and BCTs employed^a^

Study	Setting (delivered by)	Intervention classification	BCT categories	Outcome
Sirard et al. [46]	School/local neighbourhood (study staff)	Active travel to school	Social support Repetition and substitution	Desirable
McKee et al. [55]	School and family (classroom teacher)	Active travel to school	Goals and planning Feedback and monitoring Shaping knowledge	Desirable
McMinn et al. [47]	School and family (classroom teacher)	Active travel to school	Goals and planning Feedback and monitoring Shaping knowledge	Undesirable
Bungum et al. [48]	School/local neighbourhood (school staff)	Active travel to school	Associations Reward and threat	Undesirable
Mendoza et al. [49]	School/local neighbourhood (study staff)	Active travel to school	Social support Repetition and substitution Comparison of outcomes	Desirable
Mendoza et al. [50]	School/local neighbourhood (study staff)	Active travel to school	Social support Repetition and substitution	Desirable
Wen et al. [51]	School/local neighbourhood (school staff, local council officers)	Active travel to school	Feedback and monitoring Shaping knowledge Natural consequences Repetition and substitution Antecedents	Desirable
Ford et al. [53]	School only (teaching assistants and researcher)	Structured exercise, walking	Social support	Desirable
Morrison et al. [57]	Family/home (parents and researcher)	Unstructured exercise, walking	Goals and planning Feedback and monitoring Social support Comparison of behaviour Repetition and substitution Comparison of outcomes Antecedents	Undesirable
Schofield et al. [54]	School only (principal researcher/research assistant)	Educational/counselling, generic	Goals and planning Feedback and monitoring Associations Repetition and substitution	Desirable
Shimon and Petlichkoff [56]	School only (physical education teachers and assistants)	Educational/counselling, generic	Goals and planning Feedback and monitoring Social support	Desirable
Lee et al. [52]	School only (researcher/nursing tutor)	Individualised counselling	Goals and planning Feedback and monitoring Social support Natural consequences Associations Repetition and substitution Reward and threat	Desirable

*BCTs* behaviour change techniques

^a^Categorised using BCT taxonomy [39]

## Discussion

This systematic review evaluated the effects of interventions aimed at promoting walking among children and adolescents (aged 5–18 years). The results indicate that promoting walking in children and adolescents may be effective in increasing levels of PA in this population. Of the 12 studies included, nine reported increases in walking as a result of the intervention.

### Summary and Interpretation of Findings

The majority of identified studies from this systematic review were delivered in an educational setting (school/college). Since this age group spends a significant proportion of their waking time within the educational setting, it is a highly suitable setting for the promotion of PA and reduces dependence on family input [58].

Seven studies focused on increasing walking through active travel to school, with five of these reporting desirable intervention outcomes [46, 49–51, 55]. These studies employed a range of different methods to assess changes in walking, making it difficult to directly compare which approaches produced the greatest changes in walking levels in children.

An active travel to school day, which was a one-off event, found no significant effect on levels of walking [48], probably because of the short-term nature of the intervention [48]. A longer intervention (6 weeks), also involving active commuting to school, observed decreases in PA at follow-up for both intervention and control groups [47]. A number of factors may have contributed to this decline, including weather and parental influence on choice of travel to school, coupled with a lack of intervention fidelity [47].

Although the studies included within the present review often did not include health outcomes within their study design, a previous review on the association between active travel to school and health-related fitness found this form of PA was associated with healthier body composition outcomes and improved levels of cardiorespiratory fitness in young people [45]. Furthermore, the present review focused solely on walking and thus interventions which also included cycling as a form of active transportation were not included. Based on the available evidence, active travel to school by walking may contribute to increased levels of walking in children but this effect may be dependent upon environmental factors and parental decision making.

A smaller number of studies (*n* = 3) targeted at adolescents included intervention content that incorporated the provision of unsealed pedometers with individualised [52] or group discussions [54, 56]; all of these studies reported desirable intervention outcomes. No walking interventions in adolescents focused on active travel to school as a main intervention component, which is in contrast to the majority of studies targeted at children. Although there is a general consensus that PA levels decline as children move into adolescence, there is inconsistency within the literature about the context of this decline in PA, particularly as children transition from primary to secondary education, and towards adolescence. Studies have reported increases in active travel amongst boys and girls after this transition [59], while evidence in girls has shown that rates of active travel to school (walking/cycling) have been shown to remain constant, and not increase, as girls move to secondary school [60]. In contrast, longitudinal data has shown increases in the likelihood of active travel to school until the age of 10 years, when the likelihood begins to decrease [61]. Given that walking to school can contribute up to 34 % of total daily MVPA in children aged 11–12 years [62], the potential for walking to school to increase PA should be further examined by practitioners and policymakers alike as a key approach to tackling the problem of inactivity, particularly amongst adolescents [62].

School-based interventions are known to be important for the promotion of PA levels [63]; however, there is a need for further evaluation of such interventions to assess the long-term benefits on levels of PA [63]. In the present review, the majority of studies did not include a longer-term follow-up post-intervention, making it difficult to draw conclusions on the longer-term effects of such interventions on walking levels. Short-term changes in behaviour are feasible through the implementation of effective interventions [64], with the literature suggesting that the shorter-term nature of interventions may play a role in maintaining the interest and motivation of participants, particularly children [64]. However, similar to the present review, less is known about how effective such interventions are at maintaining changes in PA behaviour.

Parental involvement may form an integral component of school-based interventions [63], with strong evidence indicating that interventions involving schools in combination with family members could increase PA in adolescents [34]. Only one school-based study reporting desirable intervention outcomes involved family members as part of the intervention [55]. Parental support and direct help in facilitating PA have been shown to positively correlate with PA in adolescents [21]; however, in the present review, parental involvement was not outlined in the included studies targeted at adolescents. In addition to social support from parents, peer relationships can play a role in PA involvement at this stage of the lifecycle [65]; however, only one study explicitly mentioned participating in walks with friends as a means of increasing walking behaviours [56].

Targeting PA interventions to sub-groups of the population remains a key focus for research as young people transition from childhood into adolescence. Although ten included studies were targeted at both sexes, only two of these studies provided comparisons between sexes on intervention outcomes [48, 56]. Shimon and Petlichkoff observed no differences between sexes for daily steps post-intervention [56]. An active travel day did not result in desirable intervention outcomes for walking; however, significant differences were observed between sexes for active travel to school, with girls significantly increasing their active travel to school compared with boys, with walking cited as the most common mode of active travel [48]. The lack of evidence on the effectiveness of interventions in relation to sex limits conclusions on how best to promote walking amongst sexes.

Given that girls show a greater decline in PA than boys [14], this may be a suitable target subgroup for walking interventions. Within this review, two studies targeted girls only [52, 54], with both achieving increases in PA. These interventions did not involve structured walking sessions or walking school buses; instead participants were encouraged to set goals and increase their levels of walking by changing smaller behaviours [52, 54]. Given the preferences of adolescent girls, in particular those who are least active, for activities that are non-competitive and informal in nature [29], such intervention content may represent a more suitable means of increasing walking in this population. Furthermore, given that girls are less likely to be active during school recess periods [66], coupled with a decline in PA during recess as girls move towards adolescence [60], other opportunities to promote walking outside of active travel should be examined; for example, opportunities to walk around school grounds has been highlighted as a potential means of increasing walking and subsequent PA [67]. A meta-analysis of interventions to increase PA in young people (<11 years old) highlighted that larger effects were found for studies that were targeted at girls only [64]. Given the limited number of studies considered within this review, it is not possible to determine whether interventions that were sex-specific or mixed sex were more effective in relation to walking levels.

A number of studies within this review aimed to increase walking via multiple small bouts throughout the day rather than a single longer bout. Accumulated walking (in smaller bouts) has been shown to be as effective at increasing PA in adults as walking for a single, longer bout [68]. This may represent a more feasible means of increasing PA within paediatric populations where spontaneous PA is known to be intermittent rather than continuous in nature [69]. Adverse outcomes as a result of participating in walking were not identified within included studies, suggesting that walking may present a low risk of injury and increase its acceptability as a form of PA.

BCTs employed within interventions were multiple and varied. Social support, repetition and substitution, feedback and monitoring and goals and planning were the most frequently employed BCTs within interventions producing desirable outcomes on walking. Successful active travel interventions in children incorporated social support alongside other BCTs, suggesting that the support of others (e.g. researchers, parent volunteers and other children) may be important in the adoption and maintenance of walking to school over car use or other methods of transport. The three interventions aimed at adolescents employed similar BCTs, namely goals and planning, and feedback and monitoring, suggesting that such techniques may be effective at eliciting changes in walking levels in this age group. Pedometers have been shown to increase awareness of how behavioural choices impact upon subsequent PA by providing live feedback, which can assist individuals in making changes to increase their PA [44]. The provision of pedometers for self-monitoring, coupled with individualised or group information sessions, may therefore be effective at increasing walking in adolescents although there is a need for further studies in this age group. Such techniques may be more suitable for adolescents, who possess greater cognitive skills to employ and utilise these techniques, than children. A previous review of BCTs to promote walking and cycling in adults highlighted that self-monitoring and intention formation may be effective tools in promoting these PA behaviours [40]. Although self-monitoring was also identified in the current review as a potential BCT for inclusion in future interventions, there is a lack of evidence on how the use of BCTs translates from adult studies to children [70]. The identification of feedback, monitoring, and goals and planning as potentially successful intervention components to promote PA in children and adolescents within the present review is in contrast to the BCTs identified as effective in the management and prevention of obesity in children [70]. Techniques including prompt practice, environmental restructuring and the provision of information on individual consequences of a behaviour were recommended for use in future obesity prevention/management interventions [70]; however, these BCTs were not identified within the present review. From the available data, incorporating BCTs may be effective in combination with other intervention components in increasing walking behaviours in both children and adolescents.

Of the nine studies that reported a desirable outcome in relation to walking behaviours, seven used an objective measure to assess walking levels. Three studies used sealed pedometers, with information downloaded by study personnel rather than relying on participants to self-report their daily steps [52, 54, 56], reducing the chance of participants misreporting steps and tampering with the devices. The use of pedometers for measurement, as well as an intervention technique to facilitate self-monitoring, has been cited as a potential confounder in studies [54]. While pedometers are beneficial at detecting increases in steps over an intervention period, they do not enable researchers to measure the intensity of activities performed [44]. Despite observed increases in daily steps, it is therefore unclear if such increases were at a sufficient level of intensity to meet current MVPA guidelines and receive the associated health benefits. Two studies that used accelerometers demonstrated that increased walking contributed to increased levels of MVPA in children [46, 50]; however, the limited number of studies makes it difficult to draw associations on the contribution of walking to levels of MVPA. Previous work has highlighted the contribution walking to school can have on levels of moderate intensity PA [32], with validation studies suggesting brisk walking is sufficient to achieve moderate intensity PA in children and adolescents [71, 72].

The level of randomisation varied within studies from individual to class- and school-level randomisation. Randomising by school may be problematic as the chance of school-level factors such as a special event confounding upon results is increased. On the other hand, recruiting both intervention and control participants from the same schools [46, 52, 53, 56] may have introduced contamination with children, parents and teachers communicating with each other about intervention content. Within these studies, two interventions reported increases in walking levels for both intervention and control groups at follow-up [53, 56]. Although increases in control groups were not significant, such findings may suggest that the school environment is a fertile ground for spreading messages and promoting PA.

### Strengths and Limitations of this Review

This is the first systematic review to focus on the impact of interventions aimed at promoting levels of walking in children and adolescents. Although RCTs are regarded as the gold standard in intervention design, this review included other study designs to ensure that all available evidence was captured. Lack of randomisation decreases the validity of a study but can provide an important insight into which intervention components may or may not work when designing programmes for a given population.

This systematic review has some limitations. The first of these was the small number of identified studies eligible for inclusion. Due to limited resources, only papers published in English were eligible for inclusion. In order that the effect of the intervention on walking could be determined in isolation, this review focused only on studies that had reported changes in walking behaviours. Other interventions that employed walking in combination with other activities, such as cycling and running, were not included as data for walking alone was either unreported or unpublished. It is also acknowledged that the majority of studies reported desirable intervention outcomes, which may be due to publication bias [73].

### Unanswered Questions and Future Research

Given the small number of studies identified from this systematic review, it is evident that further research on the promotion of walking among children and, in particular, adolescents is warranted. Owing to sex-related differences in PA, further interventions tailored to girls are justified. From currently available evidence, it appears that walking interventions may be an effective means of increasing walking levels and subsequent PA in this population. Few studies provided a translation of walking outcome measures in relation to intensity, making it difficult to draw conclusions on the effectiveness of such interventions in helping children and adolescents move closer towards meeting current PA recommendations. Research has highlighted that walking may provide further opportunity for overweight and obese participants in relation to meeting these PA recommendations, owing to the increased intensity of the activity in this population [23]. Consequently, the effectiveness of walking interventions in overweight/obese populations warrants further study. In addition, only one study was identified that examined the effectiveness of walking interventions outside of the school setting; this may present opportunity for further research in other/novel settings.

## Conclusions and Implications

This systematic review highlights that walking interventions may provide an effective means for increasing walking behaviours in younger populations, at least in the short term. The majority of school-based walking interventions were shown to be effective at increasing walking in both children and adolescents. Specifically, active travel to school interventions have been shown to increase levels of walking in children; however, a lack of studies in adolescents has been highlighted, which may represent a possible focus for future policy in relation to the promotion of active travel to school, particularly within secondary schools. Such findings have implications for those involved in the promotion of PA in this age group. Schools/ teachers can play a key role in providing further opportunities for walking within the school environment, in addition to active travel. Furthermore, this review has highlighted the importance of targeting interventions, either by age or sex. This review has identified, for the first time, a number of BCTs that may be effective in promoting walking in this population and that should be utilised by practitioners working to promote PA and included in future interventions to fully assess their effectiveness. The limited number of studies to date makes it difficult to draw conclusions on the effectiveness of walking interventions in relation to different ages, sex, ethnic or socioeconomic backgrounds. Furthermore, this review has highlighted areas for future research needed to provide evidence in relation to walking and PA in children and adolescents.

## Electronic supplementary material

Supplementary material 1 (PDF 14 kb)
