# Supplementary material for: Do Interventions to Increase Walking Work? A Systematic Review of Interventions in Children and Adolescents
Source: Sports Med. 2015 Dec 1;46:515–30. doi: 10.1007/s40279-015-0432-6 (PMC4801983; doi:10.1007/s40279-015-0432-6)
Supplement: Supplementary file 1 — Supplementary material 1 (PDF 14 kb) [file 40279_2015_432_MOESM1_ESM.pdf]

ELECTRONIC SUPPLEMENTARY MATERIAL

Electronic Supplementary Material, Table S1. Example of search strategy in Medline OVID

#	Searches
**1**	exp Walking/
**2**	exp Gait/
**3**	exp Locomotion/
**4**	(activ* adj3 commut*).mp.
**5**	(activ* adj3 travel*).mp.
**6**	(activ* adj3 transport*).mp.
**7**	stair*.mp.
**8**	pedometer.mp.
**9**	walk.mp.
**10**	1 OR 2 OR 3
**11**	4 OR 5 OR 6 OR 7 OR 8 OR 9
**12**	10 OR 11
**13**	exp Motor Activity/
**14**	exp Exercise/
**15**	13 OR 14
**16**	encourag*.mp.
**17**	impact*.mp.
**18**	intervention*.mp.
**19**	increas*.mp.
**20**	program*.mp.
**21**	project*
**22**	promot*
**23**	stud*
**24**	trial*
**25**	16 OR 17 OR 18 OR 19 OR 20 OR 21 OR 22 OR 23 OR 24
**26**	exp Adolescent/
**27**	exp Child/
**28**	exp Schools/
**29**	exp Students/
**30**	young*.mp.
**31**	college*.mp.
**32**	exp Universities/
**33**	26 OR 27 OR 28 OR 29 OR 32
**34**	30 OR 31
**35**	33 OR 34
**36**	12 AND 15 AND 25 AND 35
